# Exploring parental mediation trajectories and their associations with mental health and autonomy in adolescents: A latent transition analysis

**DOI:** 10.1177/14614448251391731

**Published:** 2025-11-20

**Authors:** Sophie Mayen, Anne-Linda Camerini

**Affiliations:** Università della Svizzera italiana, Switzerland; University of Vienna, Austria; Università della Svizzera italiana, Switzerland

**Keywords:** Autonomy, latent transition analysis, mental health, parental mediation

## Abstract

Longitudinal research on the characteristics and dynamics of parental mediation strategies and their associations with adolescents’ mental health and autonomy is scarce. Using three-wave panel data from 717 Swiss adolescents (collected between spring 2018 and autumn 2020), we applied Latent Transition Analysis to identify distinct parental mediation classes and adolescents’ transitioning patterns, resulting in the: *Enforcing & Engaged Approach; Hands-off Approach*; and *Moderate Approach*, showing fluctuation over time. Regression analysis revealed no significant associations between the three classes and mental health; however, adolescents in the *Moderate Approach* class reported lower perceived autonomy compared to those in the *Enforcing & Engaged Approach* class. These findings highlight that (1) mediation strategies likely reflect broader parenting styles, (2) adapt to developmental needs, and (3) high parental engagement in setting boundaries coupled with active dialogue supports autonomous adolescent development. This insight can guide caregivers, healthcare, and policy development.

The rapid digitization has led to the continued integration of digital media into almost every aspect of adolescents’ lives. According to Self-Determination Theory (SDT; Ryan and Deci, 2000), well-being and healthy development stem from three basic psychological needs: autonomy, competence, and relatedness. This theory provides a foundation for understanding how digital media can nurture or undermine these needs.

During adolescence, digital media—primarily social media—can promote self-governance, support decision-making, and aid the development of a sense of self, all driving autonomy (West et al., 2023b). They also provide adolescents with means of social connectedness and relatedness (Marciano et al., 2023) or facilitate the cultivation of competencies related to skills development or personal growth (West et al., 2023a).

Despite this, digital media can negatively impact adolescents’ health and well-being through mechanisms including addictive use (Cai et al., 2023), interference with sleep (Brautsch et al., 2023), evolutionary mismatches resulting from biased social cues (Lim and Tan, 2024), and exposure to online risks such as hate speech (Bührer et al., 2024), sexting (Mori et al., 2019), or self-harm-related content (Scherr, 2022).

Adolescents are vulnerable to critical physiological and psychological changes (Sawyer et al., 2018). They are sensitive to peer evaluations, tend to engage in risk-taking behaviors, and experience heightened conflictual relationships with their parents as they transition toward adulthood (Ciranka and Van Den Bos, 2021). These vulnerabilities may be exacerbated by the use of digital media, such as through risky online behaviors or exposure to harmful content.

Within this period, parents remain one of the most important socialization agents (Schroeder and Mowen, 2014), shaping adolescents’ psychological adjustment, media practices, and overall development.

A widely applied conceptual framework is Baumrind’s (1991) typology of parenting styles, providing a conceptual basis for understanding how parents guide their children’s media use. Parenting styles are defined by two main dimensions: demandingness (control, supervision, boundary-setting) and responsiveness (warmth, support, attunement). Crossing these yields four styles: authoritative (high warmth/high control), authoritarian (low warmth/high control), permissive (high warmth/low control), and laissez-faire (low warmth/low control) (Baumrind, 1991; Kuppens and Ceulemans, 2019). Prior studies consistently linked authoritative parenting with the most favorable adolescent outcomes (Pinquart, 2016).

Hence, parenting styles set the broader emotional tone for how parents regulate children’s digital media use and adopt strategies that express these broader styles. Parental mediation refers to strategies parents apply to manage their children’s media use, prevent negative media effects, and promote positive opportunities for digital media use (Livingstone et al., 2017). For example, authoritarian parents may rely on heavily restrictive strategies, authoritative parents may combine rules with warmth and active discussion, and laissez-faire parents may engage minimally in mediation (Tan et al., 2025).

Parental mediation theory originated in the 1980s within television research, describing strategies of restrictive mediation (rules and boundary-setting), active mediation (discussion), and co-viewing (Clark, 2011). Co-viewing, once central, has become less relevant with the privatization and individualization of media use through smartphones and individual screens, giving way to supervision strategies where parents monitor (e.g. staying nearby) without co-using content (Nikken and Jansz, 2014). Parents face new challenges in the digital era today, such as diverse platforms and personalized algorithms (Jiow et al., 2017). Accordingly, new strategies such as Internet safety mediation have been added, focusing on guiding children’s safe online navigation (Livingstone et al., 2017).

This study examines various mediation strategies in the digital context, emphasizing the importance of understanding how parental mediation adapts to adolescents’ media use. Most prior research on parental mediation has been variable-centered, focusing on single strategies in isolation and assuming uniform effects across populations (Meyer and Morin, 2016). This approach, however, often produces mixed findings because, in practice, parents combine strategies in patterned ways shaped by adolescent development (Beyens et al., 2019) and broader parenting styles (Warren and Aloia, 2019).

In addition, the cross-sectional data underlying many studies’ findings give room for contrasting interpretations as they do not account for the dynamic nature of parental mediation, which likely changes over time.

In response, we adopt a person-centered approach, applying Latent Transition Analysis (LTA; Nylund-Gibson et al., 2023) on longitudinal panel data collected from students in Italian-speaking Switzerland during their formative years. With this, we investigate gaps in our understanding of parental mediation trajectories and their impact on adolescents’ mental health and autonomy in the context of digitization. A person-centered approach captures constellations of mediation strategies, identifying subgroups of parents who apply distinct patterns (Hickendorff et al., 2018). This approach allows us to (1) identify and characterize qualitatively distinct patterns of parental mediation strategies and interpret them as proxies for broader parenting styles (Ren and Zhu, 2022), and (2) model their stability and change (i.e. transitions) over time as adolescents claim and gain greater independence.

## Effectiveness and challenges of parental mediation in adolescents’ digital media use

A large body of research has examined specific parental mediation strategies and their effectiveness, producing mixed and at times inconclusive evidence.

Meta-analytic studies suggest that restricting youths’ digital media use (e.g. via time limits, situational restricted use, or specification of permissible content) is beneficial in limiting the overall amount of time spent with media, compared to other forms of mediation (Chen and Shi, 2019). However, setting inadequate, strict rules and boundaries that adolescents perceive as overly strict or inconsistent with the broader parenting style are not without flaws: when parents usually adopt a laissez-faire style but out of the blue impose restrictions, adolescents may view these rules as arbitrary or illegitimate. That is, parental mediation practices need to be accepted, or at least tolerated, by children and adolescents to be effective (Lim et al., 2023).

Otherwise, adolescents may exhibit reactance effects or backlash mechanisms (Valkenburg et al., 2013), a phenomenon observed in research as the “forbidden fruit” or “boomerang” effect (Meeus et al., 2018: 281). For instance, in a motivational state of reactance, adolescents may secretly extend their screen time or intentionally engage with forbidden content (Valkenburg et al., 2013).

In contrast to restrictive mediation, active mediation encourages mutual exchange about media consumption (Shin and Lwin, 2022), including agreements on usage rules or parents openly sharing their concerns about their child’s privacy. Studies on the effectiveness of active mediation show similarly mixed outcomes: Active mediation (e.g. talking with children what they are doing online, asking if they need help, showing how to protect personal information online, discuss about how to respond to online risks such as bullying or harassment; Livingstone et al., 2017) has been proven less effective than restrictive mediation in terms of limiting screen time but more effective in reducing risks related to digital media use (Chen and Shi, 2019). Other studies found active mediation related to mitigating aggression, substance use, and sexual behavior (Collier et al., 2016) and problematic Internet use (Fam et al., 2023).

This situation creates a dilemma for parents: On the one hand, they often want to set clear rules for media use; on the other hand, these rules and their monitoring need to be perceived as appropriate for adolescents. Furthermore, restricting screen time does not necessarily protect from exposure to detrimental content (Przybylski and Weinstein, 2017).

Three main factors likely account for the inconsistencies in the research regarding the effectiveness of parental mediation strategies: First, the increasingly complex digital media environment may challenge parents to effectively use established mediation strategies to manage their child’s media use (Jiow et al., 2017). Fam et al. (2023) found that parents often struggle to monitor adolescents’ digital activities, feeling frequently outmaneuvered by their children in discussions or boundary-setting attempts. Second, parental mediation strategies are likely not static but dynamically applied and influenced by various contextual factors—reflecting an ongoing reorientation of mediation approaches and a dynamic interaction between parental guidance or surveillance and adolescents’ growing autonomy.

As children grow older, parents typically reduce restrictive strategies and adopt more autonomy-supportive, active forms of guidance, reflecting adolescents’ increasing independence and need for autonomy (Opgenhaffen et al., 2012). For example, Beyens et al. (2019) noted a curvilinear pattern in parental mediation, peaking in middle childhood before declining through adolescence. This decrease is particularly noticeable as parents of younger children are more likely to impose restrictions, while parents of older adolescents tend to adopt more active forms of mediation and lessen the extent of restrictions (Nagy et al., 2023).

Third, effectiveness likely depends on the strategy itself and how it aligns with the broader parenting style (Warren and Aloia, 2019). The ways parents combine different strategies can serve as indicators of these.

To examine this, we employ a person-centered approach to identify distinct patterns of parental mediation and track their stability or changes over time. We ask:

*RQ1.* What are the distinct latent classes of parental mediation that can be identified What are the distinct latent classes of parental mediation that can be identified over time, and how are they characterized?
*RQ2.* As adolescents age, does their membership to the identified classes of parental mediation remain stable or do they transition, that is, what are the patterns of observed changes?

## Parental mediation and adolescents’ mental health and autonomy

Comprehending the varied patterns of parental mediation as specific expressions of broader parenting styles is important, particularly regarding their potential repercussions on adolescents’ mental health and autonomy.

Mental health is “a state of mental well-being that enables people to cope with the stresses of life, realize their abilities, learn well and work well, and contribute to their community” (WHO, 2022). As such, mental health comprises hedonic and eudaemonic well-being that, respectively, describe feelings of happiness and a sense of meaning and self-realization. The latter are closely linked to fundamental psychological needs, that is, competence, autonomy, and relatedness (SDT; Ryan and Deci, 2000).

Autonomy in this framework refers to volitional functioning or intrinsic motivation, meaning that adolescents perceive their behavior as self-endorsed and aligned with their values (Ryan and Deci, 2000, 2017). This differs from the autonomy-as-independence concept, which emphasizes parental separation and self-reliance. Autonomy-as-volition, supported, for example, when parents acknowledge adolescents’ viewpoints and provide rationales for rules, consistently links to well-being, whereas autonomy-as-independence shows mixed associations with adolescent adjustment (Beyers et al., 2024).

Mental disorders affect many youths, with underdiagnosis and undertreatment considered a global public health problem. The prevalence of mental health symptoms, including depression and anxiety, doubled during the COVID-19 pandemic (Racine et al., 2021), and deteriorated symptoms remained high afterward (Wang et al., 2022).

At the same time, youths’ digital media use has exponentially increased, prompting concerns about its potential impact on mental health (Twenge et al., 2020). Consequently, parental mediation has been extensively studied as a buffer against media-related risks such as exposure to pornographic or violent content (Rasmussen et al., 2016), cyberbullying victimization (Wright, 2018), and problematic Internet use (Lukavská et al., 2022).

Restrictive mediation is often considered the most effective strategy for reducing and managing screen time, which may in turn help to protect adolescents from adverse mental health outcomes related to excessive use, such as poor sleep. However, the relationship is complex: While rules on usage duration can reduce online risks, Chen and Fan (2024) found that time-bound rules may increase problematic Internet use among adolescents, whereas content restrictions are more effective in reducing it. One possible explanation for the inconsistencies among studies evaluating the effectiveness of restrictive mediation efforts is weak parent–adolescent communication, for example when adolescents withhold information, parents impose rules without context, or there is little open discussion of problems and concerns (Keijsers and Poulin, 2013).

In addition, adolescents may engage in risky online behaviors to assert their autonomy when they perceive rule-bound mediation strategies as overly controlling (Meeus et al., 2018)—a response that may undermine the effectiveness of restrictive mediation in preventing adverse mental health outcomes related to media use (Padilla-Walker et al., 2020). Furthermore, stricter, and less open communication with parents can make adolescents less likely to seek parental support, leaving them more vulnerable to internalizing problems. Finally, the previously mentioned reverse causality might explain why restrictive mediation is linked to worse mental health. In this case, parents react to digital media-related problems in their children with time and content restrictions (Geurts et al., 2024).

The emerging pattern suggests that an increase in, or consistently high levels of, stricter and rule-heavy mediation could negatively impact adolescents’ mental health. We anticipate a variety of mixed mediation strategies in the digital environment. Identifying and characterizing these within our analyses as latent classes of parental mediation and how these change as adolescents age remains subject to our exploratory analyses. Consequently, we pose a third research question to generate hypotheses for future studies regarding the effects of parental mediation on mental health:

*RQ3.* What is the relationship between the identified latent classes of parental mediation and adolescents’ mental health?

During adolescence, youths typically develop a strong demand to act increasingly autonomously from their parents, also in terms of their media use (Padilla-Walker et al., 2020). Restrictive mediation holds strong potential to undermine adolescents’ autonomy: Imposing and enforcing strict rules leaves little room for youths’ individual development of (media) autonomy (Livingstone et al., 2017).

Active mediation strategies indicate a supportive parent–child bond, marked by positive parenting and higher involvement (Lim et al., 2023), likely indicative of autonomy-supportive parenting approaches. In this sense, active mediation is more likely to promote adolescents’ autonomy needs as this strategy values their perspectives and fosters autonomous self-regulation (Meeus et al., 2018).

While the precise classification of parental mediation classes is subject to the analysis of this present study, we anticipate discovering overarching patterns that contrast strict, rule-bound approaches with more liberal, discussion-oriented, or laissez-faire practices. Depending on the distinct latent classes of parental mediation identified through LTA and their observed changes over time, we will proceed to test the following hypotheses:

*H1a.* An increase in or constant stricter (i.e. rule-based) parental mediation will be associated with lower perceived autonomy among adolescents.
*H1b.* An increase in or constant liberal (i.e. discussion-oriented) parental mediation will be associated with higher perceived autonomy.

## Methods

### Study design and sample

The study was pre-registered on OSF (https://osf.io/w7nms/?view_only=5f049b701516477daea7ea8f3d6b0582) and used data from the MEDIATICINO project, a longitudinal study conducted in Switzerland between 2014 and 2021 that followed students annually as they transitioned from childhood into adolescence to investigate the interplay between digital media use and well-being (https://mediaticino.usi.ch/en). Participants comprised approximately one-third of the underlying population, representative of adolescents born in 2004/5. For this study, data were drawn from waves 5 to 7 (T1 to T3), collected through a paper-and-pencil questionnaire at school in the spring of 2018 (T1), 2019 (T2), and in the autumn of 2020 (T3). Participants were randomly selected at the class level at the study’s onset in 2014. Their annual questionnaire responses were matched with the help of a unique identifier provided by the regional education administration (for a detailed description of sampling and questionnaire administration see Camerini et al., 2018). The project adequately addressed ethical considerations regarding anonymity and voluntary participation. It was approved by the regional education administration.

### Measures

*Parental mediation* was repeatedly measured at T1, T2, and T3 using 19 items adapted from previous studies (Dürager and Sonck, 2014; Hiniker et al., 2016; Livingstone et al., 2017; Nikken and Jansz, 2014). For a list of all parental mediation items, see Supplemental Appendix A. They covered restrictive mediation, active mediation (including Internet safety mediation) and supervision of digital media use. The items were randomly listed in a single block that was introduced with the sentence: “During the past year, with respect to the use of computers, tablets, or smartphones, at least one of my parents . . .” Examples include “. . . adjusted the duration of use” (*restrictive mediation*; Hiniker et al., 2016); “. . . suggested how to use the Internet safely” (*active/Internet safety mediation*; Livingstone et al., 2017); “. . . ‘glanced over’ while I was using devices” (*supervision*; Nikken and Jansz, 2014). Each item was measured on a 5-point scale ranging from 1 (“never”) to 5 (“always”), with higher values indicating more frequent use of the respective mediation strategy.

*Mental health* was assessed at T3 using seven subdomains from the DSM-5 Cross-Cutting Symptoms Measure for individuals aged 6–17 (Clarke and Kuhl, 2014). These subdomains cover *somatic symptoms*, that is, “Been bothered by stomach aches, headaches, or other aches and pains,” *sleep problems*, that is, “Been bothered by not being able to fall asleep, stay asleep, or waking up too early,” *inattention*, that is, “Been bothered by not being able to pay attention in class, during homework, reading, or playing,” *obsessive-compulsive disorder* (OCD), that is, “Feeling the need to do certain things repeatedly (e.g. washing hands, checking, tidying up),” *anger*, that is, “Felt angry or lost your temper,” *irritability*, that is, “Felt more irritated or easily annoyed than usual,” and, finally, *anxiety*, that is, “Felt nervous, anxious, or scared”; “Not been able to stop worrying.” Each item was measured on a 5-point scale ranging from 1 (“never”) to 5 (“almost every day”). The overall mental health score was calculated as the mean of reverse-coded subdomain scores (*M* = 3.56, *SD* = 0.74), with higher values indicating better mental health.

*Perceived autonomy* was assessed at T3 using a set of 10 items (adapted from Noom et al., 1999). The items covered functional aspects of decision-making (e.g. “I easily decide what I want”) and relational aspects (e.g. “When I’m with my friends, I know how to make my point”). As such, the final measure captures autonomy’s volitional aspect, consistent with SDT’s conceptualization of self-endorsed, value-congruent behavior (Ryan and Deci, 2000). Three reverse-coded items were excluded from the final analysis because they continued to negatively correlate with other items even after statistical reverse coding, suggesting potential misunderstanding by respondents. The remaining items were scored on a scale from 1 (“strongly disagree”) to 7 (“strongly agree”), with higher values indicating higher levels of autonomy (*M* = 4.6, *SD* = 1.11; Cronbach’s α = 0.82).

*Covariates* include *gender* measured as 0 (“male”) and 1 (“female”), *age* (continuous; see Table 1 for average at each wave), and *socioeconomic status* measured with a single item: “How well off do you think your family is?” on a scale from 1 (“very well”) to 4 (“not really well”). The scale was reversed and collapsed into low, medium, and high, using predefined cut-off points for sample description. Covariates further include *device ownership* measured as 0 (“no”) and 1 (“yes”) for smartphone, tablet, and laptop/PC with Internet. These covariates were continuously assessed each year.

**Table 1.** Sample characteristics.

	T1			T2			T3		
	*M*	*Md*	*SD*	*M*	*Md*	*SD*	*M*	*Md*	*SD*
Age	13.5	13	0.57	14.4	14	0.55	16.1	16	0.41
Gender (%)									
Male	44.9								
Female	55.1								
Socioeconomic status (%)									
Low	26.6			29.8			38.6		
Medium	58.7			56.2			51.2		
High	14.6			13.9			10.2		
Device ownership* (%)									
Smartphone	92.9			96.8			99.6		
Tablet	56.1			46.4			39.2		
Laptop/PC	52.9			70.9			90.0		

*Note. N* = 717. *M* = mean; *Md* = median; *SD* = standard deviation; *with Internet.

Finally, we measured *social desirability* at T3. We used eight items from the Children’s Social Desirability Short (CSD-S) scale (Camerini and Schulz, 2018). An example item is: “Have you ever felt like saying unkind things to a person?” Answer options ranged from 1 (“never”) to 5 (“always”). All items were reverse coded so that higher values indicate higher levels of social desirability responding (*M* = 3.50, *SD* = 0.66; Cronbach’s α = 0.83).

### Analytical strategy

We conducted the analysis on adolescents who participated in all three waves considered in this study. We imputed missing data on single measures using the “mice” package in R (Buuren and Groothuis-Oudshoorn, 2011; Enders, 2023). Our pre-registered analytical approach involved four steps. First, we conducted latent class analysis (LCA) at three time points (T1→T2→T3) to identify subgroups of adolescents with similar patterns across 19 items measuring different parental mediation strategies. We tested models with 2 to 5 classes, including covariates, and compared model fit using AIC, BIC, aBIC, and entropy. We also visualized fit statistics with an elbow plot. Second, we specified the LTA model and generated transition probabilities. To ensure consistency in the definition of latent classes across time points, measurement invariance was imposed by constraining the item-response probabilities to be identical across T1, T2, and T3. This approach allows for the latent classes to be consistently interpreted across different waves, ensuring that observed transitions between classes can be attributed to genuine changes rather than differences in class definitions. Finally, class probabilities were extracted from the LTA model at T3, assigning adolescents to their most likely class based on the highest posterior probability. To examine the association between class membership and outcomes, we conducted separate regression analyses with mental health and autonomy as dependent variables. Regression models controlled for social desirability responding. We used the “polCA” package (Linzer and Lewis, 2011) for LCA and the “depmixS4” (Visser and Speekenbrink, 2010) package for LTA in R (version 4.3.2 [2023-10-31]). The R script for all analyses can be accessed on OSF.

## Results

### Sample

Questionnaire data was collected from 1374 adolescents at T1, 1263 at T2, and 1088 at T3. Sample attrition was mainly due to adolescents being absent on the day of questionnaire completion or moving away over the course of 3 years. The analytical sample included 717 participants who completed all 3 waves. Sample characteristics are shown in Table 1.

### Specification of parental mediation classes

We fitted LCA models with 2 to 5 classes (see Supplemental Appendix B). After inspection of fit indices for LCA models and the elbow plot shown in Figure 1, we selected a three-class solution for its appropriate minimum class proportion (14.8%), size (*n* = 106), and theoretical relevance.

**Figure 1.**
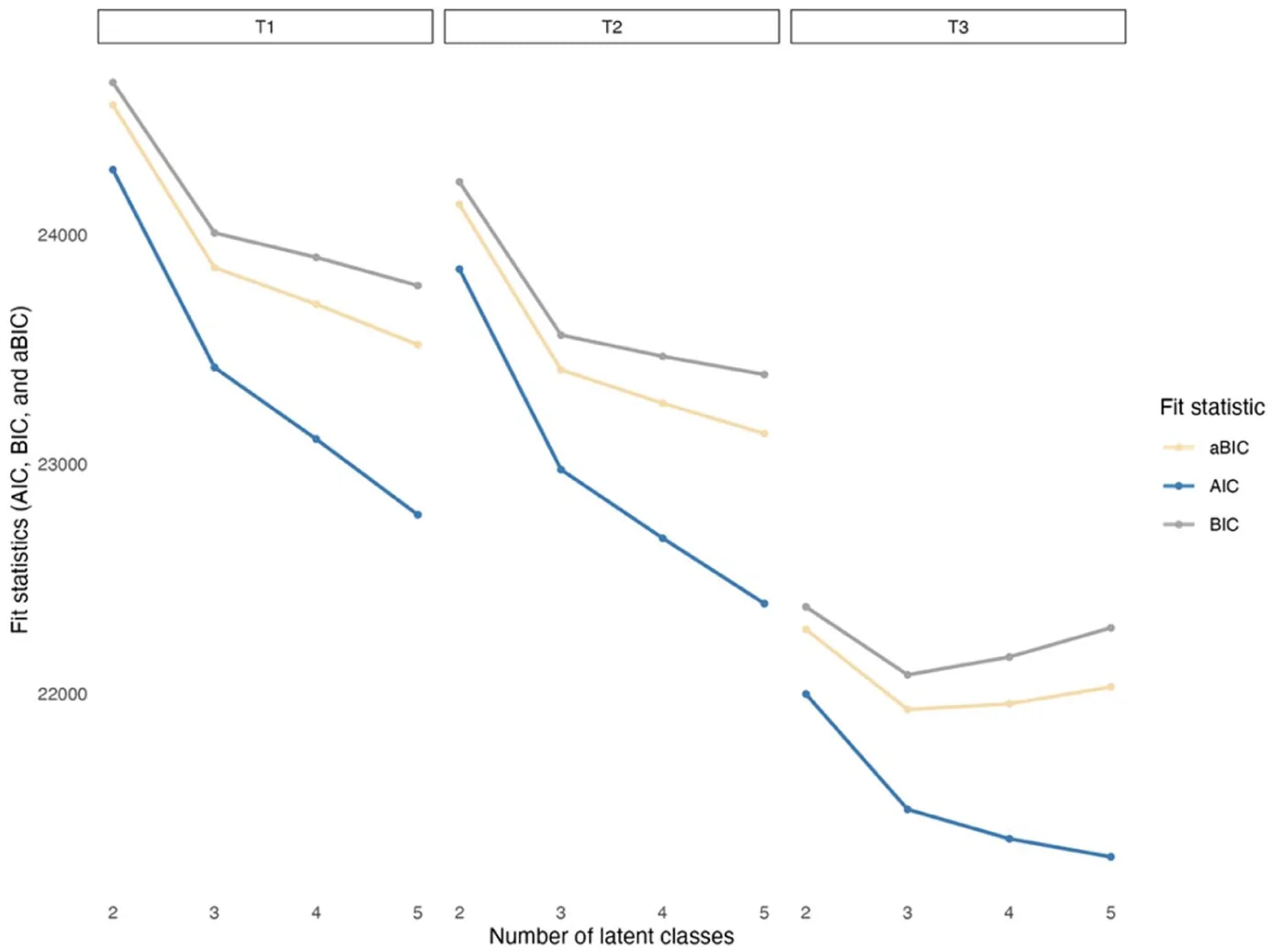
Elbow plot for model fit statistics across time points.

The identified classes (RQ1) were: (1) *Enforcing & Engaged Approach*; (2) *Hands-off Approach*; and (3) *Moderate Approach.* Posterior probabilities were extracted from the fitted LCA model to obtain the probability of each individual belonging to each of the three latent classes (see Supplemental Appendix C). At T1, 16.1% of the students belong to Class 1 (*n* = 116), 44.3% to Class 2 (*n* = 317), and 39.6% to Class 3 (*n* = 284).

Adolescents in Class 1 were subject to an *Enforcing & Engaged Approach*. They reported that parents enforced more restrictive rules on digital media use, such as limiting the duration of media use or ensuring that media is not used during certain times (e.g. during meals, before bedtime). However, adolescents in this class also perceived that their parents regularly use active mediation strategies, and they perceived high levels of supervision. They further reported that their parents implement rules to ensure Internet safety. While responses from Class 1 adolescents regarding the use of media as a reward or punishment showed a bimodal pattern—indicating either low or high levels—their parents tended to use media more frequently as a form of reward or punishment compared to parents of adolescents in the other classes.

In contrast, Class 2 adolescents experienced a *Hands-off Approach*, that is, they perceived few rules concerning their media use and low parental involvement in time management (i.e. restrictive mediation), Internet safety mediation, active mediation, and media supervision. Parental mediation in this class was generally minimal across all strategies (restrictive, active, supervision, Internet safety). Nevertheless, two responses regarding stricter rules—asking children to put away media during meals or before bedtime—again showed a bimodal pattern, suggesting that parents of students in the *Hands-off Approach* class varied in their enforcement of these situation-specific regulations.

Patterns of parental mediation in Class 3 can be best described as a *Moderate Approach*, with adolescents reporting moderate levels across diverse strategies. Parents in this class were perceived as less strict than those in the *Enforcing & Engaged Approach* class, but more involved in restrictive and active mediation types than those in the *Hands-off Approach* class. Parents tended to enforce rules around situation-specific media use (e.g. during meals, at bedtime) while maintaining moderate engagement in supervision and active mediation, including active mediation of Internet safety. Based on adolescents’ perceptions, Class 3 parents predominantly applied active mediation strategies that included discussing online activities, encouraging independent exploration, and recommending specific sites. Figure 2 displays the presence of parental mediation strategies for each class and wave.

**Figure 2.**
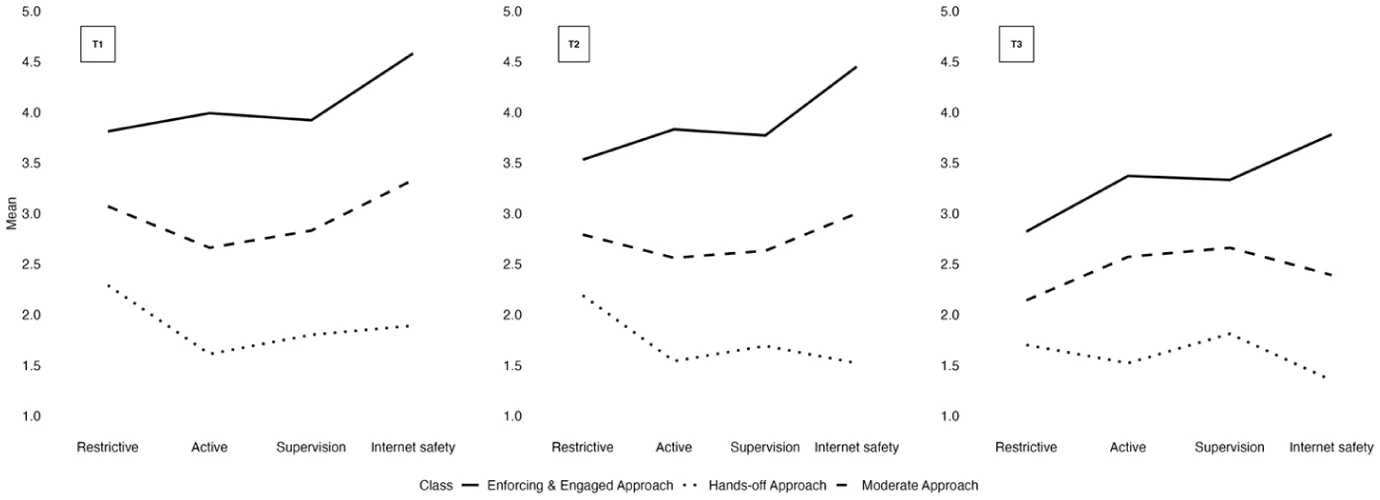
Parental mediation strategies for each class across time points.

Ongoing, RQ1 sought to assess whether the class characteristics identified at T1 remained stable or changed over time. Adolescents in the *Enforcing & Engaged Approach* class consistently perceived their parents enforcing a regulatory, restrictive, and active parental mediation approach. This pattern showed particularly strong persistence through T1 and T2. At T3, as adolescents aged, the *Enforcing & Engaged Approach* showed a slight decline, particularly evident in reduced regulation of media use duration, fewer comments on shared media activities, and less supervision. The *Hands-off Approach* class continued to be characterized by minimal parental engagement across all mediation strategies from T1 to T3, maintaining a permissive stance toward their children’s media use. In the *Moderate Approach* class, the level of restrictive mediation behaviors decreased from T2 to T3. In addition, there was a decline in active mediation, particularly in aspects related to supervision, such as parents “glancing over” while adolescents were using devices.

### Transitioning patterns between parental mediation classes

Addressing RQ2, we first examined how the class share proportions shifted across time points (see Supplemental Appendix D). The proportion of students in the *Enforcing & Engaged Approach* class remained relatively stable, slightly decreasing over time (from 16.1% at T1 to 14.8% at T3). In contrast, the share of the *Hands-off Approach* class declined between T1 (44.3%) and T2 (37.5%) before increasing again at T3 (45.8%), indicating a shift toward less engaged mediation among parents in the study sample, particularly from T2 to T3. The *Moderate Approach* class showed an increase from T1 (39.6%) to T2 (46.6%), followed by a decline at T3 (39.5%), indicating some fluctuation in the adoption of this mixed approach over the study period.

Next, we fitted an LTA model with three latent classes and covariates to estimate transition probabilities over time (see Supplemental Appendix D). The transition probabilities across T1, T2, and T3 revealed distinct patterns: From T1 to T2, individuals in the *Enforcing & Engaged Approach* class had a 55.5% likelihood of remaining in the same class, while those in the *Hands-off Approach* class showed a 30.4% probability of persistence. The *Moderate Approach* class had a 33.8% probability of stability, with a notable transition probability to the *Hands-off Approach* class (47.3%). Between T2 and T3, the probability of staying or moving to another class for the *Enforcing & Engaged Approach* remained relatively unaffected (50.1%). The *Hands-off Approach* showed comparably higher class stability (i.e. staying in this class and having less probability of moving to another class; 62.0%). From T2 to T3, the *Moderate Approach* showed considerably lower class stability (19.8%) with increased probability for adolescents transitioning to the *Hands-off Approach* class, compared to the transitioning pattern from T1 to T2 during this time interval (68.7%). Figure 3 visualizes the flow of individuals transitioning between latent classes based on their posterior assignments.

**Figure 3.**
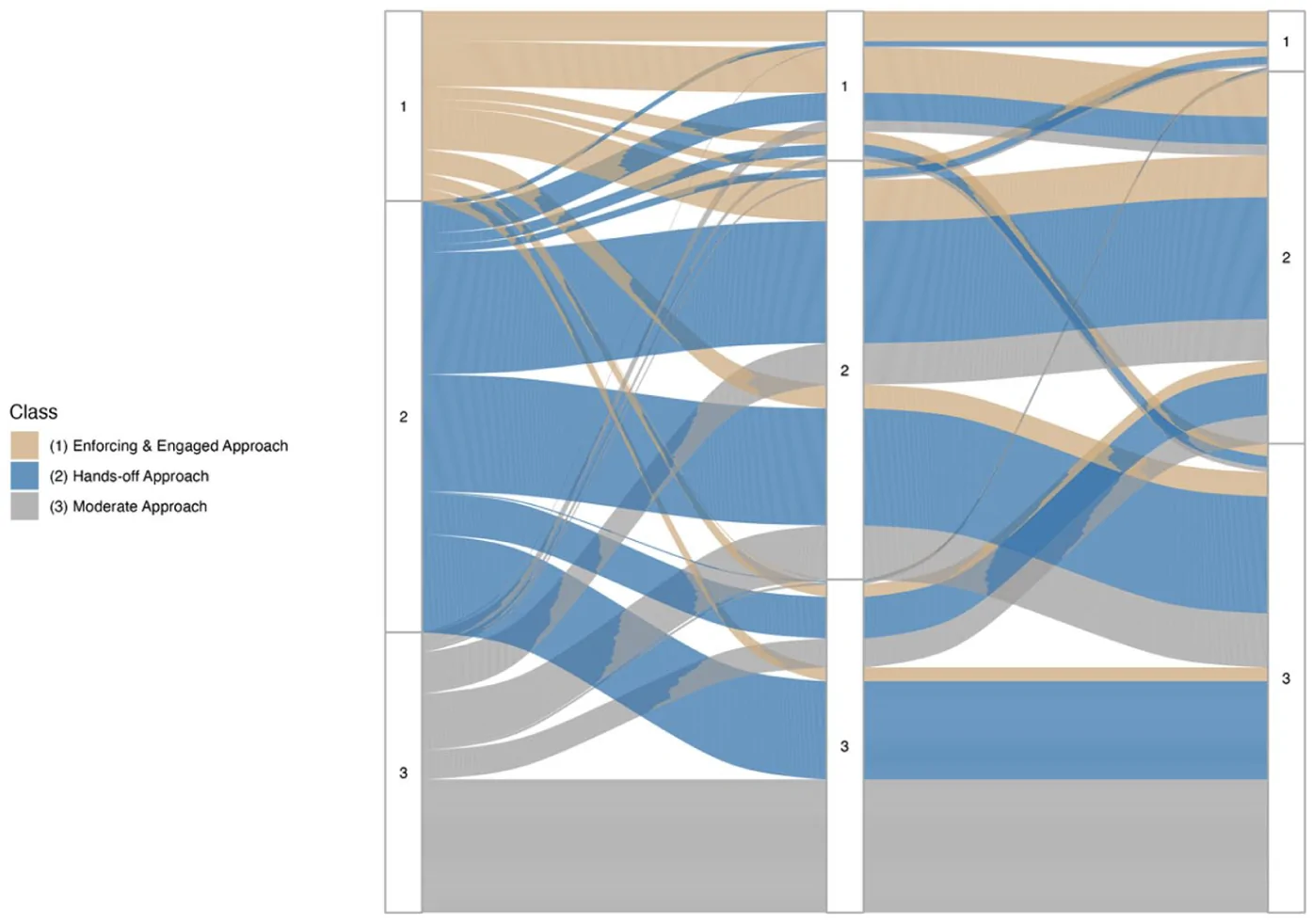
Transitions of individuals across latent classes (T1→T2→T3). *Note.* The figure depicts the class movement of individuals over time based on probability. Posterior probabilities indicate the most probable class assignments per individual at each time point, approximating class membership.

### Linear regression analysis

Regression analysis was used to predict the association between latent class membership at T3 and mental health (RQ3) and autonomy (H1a, H1b), controlling for social desirability, all assessed at T3. Table 2 provides an overview of the regression results. A supplementary regression table for the individual subdomains of mental health is available in the Supplemental Appendix E on OSF.

**Table 2.** Regression analysis (T3).

Predictors	DV: Mental health			DV: Autonomy		
	*B*	*SE*	*t*	*B*	*SE*	*t*
*RC: Enforcing & Engaged Approach*						
Intercept	2.160***	0.174	12.430	5.612***	0.277	20.286
Moderate Approach	-0.072	0.106	-0.674	-0.390*	**0.170**	**-2.297**
Hands-off Approach	-0.045	0.105	-0.428	-0.314	0.167	-1.878
*RC: Moderate Approach*						
Intercept	2.088***	0.143	14.638	5.222	0.277	22.995
Enforcing & Engaged Approach	0.072	0.106	0.674	0.390*	**0.170**	**2.297**
Hands-off Approach	0.027	0.054	0.500	0.076	0.085	0.886
*Covariate*						
Social desirability	0.417***	0.039	10.629	-0.193**	0.062	-3.094
Residual Std. Error	0.689			1.097		
R-squared	0.139			0.020		
F-statistic	38.230			4.726		

*Note. N* = 717; DV = dependent variable; *RC* = reference class: 1 = “belong,” 0 = “not belong”; *B* = unstandardized coefficient; *SE* = standard error; *t* = *t*-value; ***p < .001, **p < .01, *p < .05; significant class differences are highlighted in bold.

The model, including mental health as the outcome variable, accounted for approximately 13.9% of the variance (adjusted *R*^2^ = 0.135), and the overall model was statistically significant, *F* (3,713) = 38.23, *p* < .001. Social desirability was a significant covariate (*B* = 0.417, *SE* = 0.039, *p* < .001), suggesting that higher levels of social desirability were associated with better reported mental health outcomes, independent of class membership. Since classes were entered as dummy variables where participants were categorized as either 1 (“belong”) or 0 (“not belong”), unstandardized coefficients can be interpreted as mean differences in mental health compared to the reference class. The *Hands-off Approach* class (*B* = −0.045, *SE* = 0.105, *p* = *ns*) and the *Moderate Approach* class (*B* = −0.072, *SE* = 0.106, *p* = *ns*) did not significantly differ in their overall reported mental health compared to the reference class (*Enforcing & Engaged Approach*). Changing the reference class to the *Moderate Approach*, we found no significant difference between the *Moderate Approach* and the *Hands-off Approach* classes (*B* = 0.027, *SE* = 0.054, *p* = *ns*).

We further examined whether class membership was related to specific mental health subdomains. About the subdomain *inattention*, both the *Hands-off Approach* (*B* = −0.348, *SE* = 0.130, *p* < .01) and *Moderate Approach* (*B* = −0.340, *SE* = 0.132, *p* < .01) classes showed significantly lower levels of inattention compared to the *Enforcing & Engaged Approach* class. When setting the *Moderate Approach* as a reference class, the *Hands-off Approach* did not significantly differ from the *Moderate Approach* in reported inattention levels (*B* = −0.008, *SE* = 0.066, *p* = *ns*). No other comparison for mental health subdomains was statistically significant.

Moreover, we tested differences among the three classes of parental mediation at T3 in adolescents’ perceived autonomy at T3 (H1a and H1b), controlling for social desirability. The model accounted for a small but statistically significant portion of the variance (1.95%; adjusted *R*^2^ = 0.015), with an overall *F* (3,713) = 4.73, *p* < .01. Social desirability was a significant covariate (*B* = −0.193, *SE* = 0.062, *p* < .01), suggesting that higher levels of social desirability were associated with less perceived autonomy, independent of class membership. Compared to the *Enforcing & Engaged Approach* class (reference class), adolescents in the *Moderate Approach* class reported significantly less perceived autonomy (*B* = −0.390, *SE* = 0.170, *p* < .05). A similar picture was evident for the *Hands-off Approach* class, though the mean difference in autonomy was only marginally significant (*B* = −0.314, *SE* = 0.167, *p* = 0.061). Changing the reference class to the *Moderate Approach*, we found no significant difference between the *Moderate Approach* and the *Hands-off Approach* classes (*B* = 0.076, *SE* = 0.085, *p* = *ns*). Thus, H1a and H1b were rejected.

## Discussion

This study set out to identify longitudinal patterns of parental mediation of adolescents’ digital media use, to assess the stability and transitions among these patterns as adolescents aged, and to investigate their associations with adolescents’ perceived mental health and autonomy. We adopted a person-centered approach using longitudinal data collected over 3 years. We identified apparent class-related differences in parental mediation: Some parents consistently used the full scope of mediation strategies simultaneously (*Enforcing & Engaged Approach*), while others demonstrated minimal involvement across all mediation forms (*Hands-off Approach*). Examining these constellations, rather than distinct mediation strategies in isolation, highlights how strategies cluster in practices and thus provides a more ecologically valid picture of parental mediation. The identified classes build on and extend traditional distinctions by showing how strategies may co-occur within parenting styles. These insights help explain why evidence on parental mediation strategy effectiveness is fragmented, since outcomes likely depend on their alignment and integration within overarching parenting styles.

To note, our identified classes should not be viewed as direct equivalents of parenting styles. For example, the *Enforcing & Engaged Approach* is characterized by high levels of control and regulation, suggesting authoritarian elements, though its frequent use of active mediation and supervision also indicates parental warmth. The *Hands-off Approach* appears closer to laissez-faire or permissive styles, depending on the levels of warmth present. Finally, the *Moderate Approach* combines rule-setting and involvement, which can be viewed as characteristics of authoritative tendencies.

The identified classes remained relatively consistent over 3 years, yet the frequency of specific strategies within each class decreased gradually between ages 13 (T1) and 16 (T3), consistent with prior research on the developmental trajectory of parent-adolescent communication. As adolescents’ need for autonomy increases, parents tend to reduce their mediation efforts after having peaked in middle childhood (Beyens et al., 2019).

In examining the stability and change of parental mediation classes and their characteristics, we focused on transition patterns between classes over time. A key trend was adolescents’ gradual shift from the *Enforcing & Engaged Approach* to more moderate approaches over 3 years. Prior research has indicated that decreased rule-setting might lead to increased reactive restrictions 1 year later (e.g. Geurts et al., 2024). In contrast, our study identified no such cyclical pattern; if that were the case, we would have expected fluctuations in *Enforcing & Engaged Approach* class membership over time (e.g. transitioning to a more moderate mediation class and returning to the *Enforcing & Engaged Approach*). This difference may imply that such shifts occur on a shorter timescale. Our person-centered approach captures annual trends across the sample, yet tracking individual trajectories over shorter intervals—such as quarterly or through micro-longitudinal experience sampling—could yield additional depth.

Another important finding is that parental mediation classes were not directly linked to adolescent mental health, likely due to several factors. First, although the *Enforcing & Engaged Approach* was associated with higher perceived autonomy, mental health extends beyond this single dimension to encompass relatedness and competence; the multifaceted construct also builds upon more than the mere presence of well-being, including the absence of ill-being. Broader contextual influences on mental health (e.g. family climate, socioeconomic context) may further overshadow specific mediation strategies.

Second, prior research demonstrates that parental warmth is positively linked to adolescent mental health (e.g. Peng et al., 2021). In contrast, strict control or regulatory strategies, especially when lacking warmth, can provoke resistance in adolescents and potentially promote backfire effects (Valkenburg et al., 2013). Although our study did not directly measure parental warmth, we discuss these findings to contextualize our results within the broader parenting literature. Notably, we found no evidence that stricter forms of mediation were associated with poorer mental health in our sample. This finding extends previous work that reported such associations (e.g. Wright and Wachs, 2022).

One possible explanation is that adolescents in the *Enforcing & Engaged Approach* perceived their parents’ overall high engagement, which may have also entailed elevated levels of warmth (e.g. interpreting high engagement in mediation as signs that “my parents care for me”). This notion may buffer against potential backfire or resistance effects, but this interpretation remains speculative.

In addition, parents in the *Enforcing & Engaged Approach* and *Moderate Approach* classes frequently engaged in active mediation, suggesting that they maintained an open dialogue with their children, which may have shielded adolescents against risky online behaviors (Nielsen et al., 2019). Further research indicates that active mediation and supervision enhance digital literacy in adolescents, promoting resilience skills in managing online risks and media opportunities (Livingstone et al., 2017; Lou et al., 2024). Consequently, parental involvement in mediation strategies may equip adolescents with essential digital skills, facilitating safer and more effective maneuvering of the digital landscape.

One unanticipated finding was that adolescents in the *Moderate Approach* class reported lower perceived autonomy than those in the *Enforcing & Engaged Approach* class. Adolescents likely need boundaries to experience autonomy within a set framework. For instance, research indicated that parental regulation does not necessarily undermine adolescents’ volitional functioning. Such practices may even be beneficial during adolescence, as neuropsychological processes related to self-regulation are still maturing (e.g. Soenens et al., 2017). In contrast, parenting styles characterized by low responsiveness or high psychological control, such as authoritarian, can undermine autonomy development (Teuber et al., 2022). Nevertheless, it must be noted that we assessed autonomy in the general context in this study, not related to digital media use. Measuring autonomy and its dimensions, specifically within the digital domain, could add nuance to future investigations.

This study is subject to certain limitations. First, the reliance on self-reported data introduces potential biases, particularly due to social desirability, which we controlled for in our analyses but also significantly influenced the relationship between parental mediation class, mental health, and autonomy. Given the stigma around mental health, responses to mental health-related questions may be particularly affected. Likewise, perceived autonomy was negatively associated with social desirability, indicating that adolescents prone to please others in their responses and behaviors also exhibit lower trait autonomy, which manifests in both relational and situational contexts as captured by the measure in our study. Second, we only assessed the associations between parental mediation patterns and mental health and autonomy at T3, as not all outcome variables were available at all three waves. Investigating the longitudinal associations would provide additional insights into how parental mediation patterns evolve as a function of mental health and perceived autonomy and, *vice versa*, predict these outcomes over time.

## Conclusion

This study gives insights into the longitudinal characteristics and the dynamics of parental mediation strategies and their association with adolescent mental health and autonomy. We identified three latent classes of parental mediation in the digital environment—*Enforcing & Engaged Approach* (1); *Hands-off Approach* (2); and *Moderate Approach* (3)—that predominantly differed in parental engagement levels, likely tapping into overarching parenting styles. Modeling their latent trajectories revealed patterns of both stability and change, indicating that parental mediation strategies are context-dependent and adaptive to the developmental needs of adolescents. While class membership was not associated with mental health outcomes, this study points to potential other influences that may interact with mental health in ways not directly observed. At the same time, high parental involvement across a set of mediation strategies may promote an ideal environment for healthy autonomy development by providing structured boundaries. These findings underscore the importance of raising awareness and guiding parents through schools, pediatric care, and public health initiatives, encouraging parents to adopt approaches that balance clear rules with active dialogue. This study’s findings highlight that parent–adolescent mediation dynamics are complex, and follow-up research is needed into the interplay between parental mediation of digital media use, mental health, and the psychological development of youths.

## Supplemental Material

sj-docx-1-nms-10.1177_14614448251391731 – Supplemental material for Exploring parental mediation trajectories and their associations with mental health and autonomy in adolescents: A latent transition analysis Supplemental material, sj-docx-1-nms-10.1177_14614448251391731 for Exploring parental mediation trajectories and their associations with mental health and autonomy in adolescents: A latent transition analysis by Sophie Mayen and Anne-Linda Camerini in New Media & Society

## Data Availability

The data, R code, and supplemental material for the main analyses are available at the following link: https://osf.io/k4g7j/?view_only=44b4ac11a14c4f7eaf7ef5d82af1aa81.

## References

[bibr1-14614448251391731] Baumrind D (1991) The influence of parenting style on adolescent competence and substance use. The Journal of Early Adolescence 11(1): 56–95.

[bibr2-14614448251391731] Beyens I Valkenburg PM Piotrowski JT (2019) Developmental trajectories of parental mediation across early and middle childhood. Human Communication Research 45(2): 226–250.

[bibr3-14614448251391731] Beyers W Soenens B Vansteenkiste M (2024) Autonomy in adolescence: a conceptual, developmental and cross-cultural perspective. European Journal of Developmental Psychology. Epub ahead of print 15 March. DOI: 10.1080/17405629.2024.2330734.

[bibr4-14614448251391731] Brautsch LAS Lund L Andersen MM , et al. (2023) Digital media use and sleep in late adolescence and young adulthood: a systematic review. Sleep Medicine Reviews 68: 101742. 10.1016/j.smrv.2022.101742 36638702

[bibr5-14614448251391731] Bührer S Koban K Matthes J (2024) The WWW of digital hate perpetration: what, who, and why? A scoping review. Computers in Human Behavior 159: 108321.

[bibr6-14614448251391731] Buuren SV Groothuis-Oudshoorn K (2011) Mice: multivariate imputation by chained equations in R. Journal of Statistical Software 45(3): 1–67.

[bibr7-14614448251391731] Cai Z Mao P Wang Z , et al. (2023) Associations between problematic internet use and mental health outcomes of students: a meta-analytic review. Adolescent Research Review 8(1): 45–62. 10.1007/s40894-022-00201-9 PMC9883821 36744121

[bibr8-14614448251391731] Camerini A-L Schulz PJ (2018) Social desirability bias in child-report social well-being: evaluation of the Children’s Social Desirability Short Scale using item response theory and examination of its impact on self-report family and peer relationships. Child Indicators Research 11(4): 1159–1174.

[bibr9-14614448251391731] Chen L Shi J (2019) Reducing harm from media: a meta-analysis of parental mediation. Journalism & Mass Communication Quarterly 96(1): 173–193.

[bibr10-14614448251391731] Chen YA Fan T (2024) Adolescents’ mental health, problematic internet use, and their parents’ rules on internet use: a latent profile analysis. Computers in Human Behavior 156: 108232.

[bibr11-14614448251391731] Ciranka S Van Den Bos W (2021) Adolescent risk-taking in the context of exploration and social influence. Developmental Review 61: 100979.

[bibr12-14614448251391731] Clark LS (2011) Parental mediation theory for the digital age. Communication Theory 21(4): 323–343.

[bibr13-14614448251391731] Clarke DE Kuhl EA (2014) DSM-5 cross-cutting symptom measures: a step towards the future of psychiatric care? World Psychiatry 13(3): 314–316. 10.1002/wps.20154 PMC4219074 25273306

[bibr14-14614448251391731] Collier KM Coyne SM Rasmussen EE , et al. (2016) Does parental mediation of media influence child outcomes? A meta-analysis on media time, aggression, substance use, and sexual behavior. Developmental Psychology 52(5): 798–812. 10.1037/dev0000108 26914217

[bibr15-14614448251391731] Dürager A Sonck N (2014) Testing the Reliability of Scales on Parental Internet Mediation (EU Kids Online). London: London School of Economics and Political Science (LSE).

[bibr16-14614448251391731] Enders CK (2023) Missing data: an update on the state of the art. Psychological Methods. Epub ahead of print 16 March. DOI: 10.1037/met0000563. 36931827

[bibr17-14614448251391731] Fam JY Männikkö N Juhari R , et al. (2023) Is parental mediation negatively associated with problematic media use among children and adolescents? A systematic review and meta-analysis. Canadian Journal of Behavioural Science / Revue canadienne des sciences du comportement 55(2): 89–99.

[bibr18-14614448251391731] Geurts SM Vossen HGM Van Den Eijnden RJJM , et al. (2024) Bidirectional within-family effects of restrictive mediation practices and adolescents’ problematic social media use. Journal of Youth and Adolescence 53(8): 1928–1938. 10.1007/s10964-024-01990-z PMC11226470 38700825

[bibr19-14614448251391731] Hickendorff M Edelsbrunner PA McMullen J , et al. (2018) Informative tools for characterizing individual differences in learning: latent class, latent profile, and latent transition analysis. Learning and Individual Differences 66: 4–15.

[bibr20-14614448251391731] Hiniker A Schoenebeck SY Kientz JA (2016) Not at the dinner table: parents’ and children’s perspectives on family technology rules. In: Proceedings of the 19th ACM Conference on Computer-Supported Cooperative Work & Social Computing, San Francisco, CA, 27 February, pp. 1376–1389. ACM. Available at: https://dl.acm.org/doi/10.1145/2818048.2819940 (accessed 29 October 2024).

[bibr21-14614448251391731] Jiow HJ Lim SS Lin J (2017) Level up! Refreshing parental mediation theory for our digital media landscape: parental mediation of video gaming. Communication Theory 27(3): 309–328.

[bibr22-14614448251391731] Keijsers L Poulin F (2013) Developmental changes in parent–child communication throughout adolescence. Developmental Psychology 49(12): 2301–2308. 10.1037/a0032217 23477535

[bibr23-14614448251391731] Kuppens S Ceulemans E (2019) Parenting styles: a closer look at a well-known concept. Journal of Child and Family Studies 28(1): 168–181. 10.1007/s10826-018-1242-x PMC6323136 30679898

[bibr24-14614448251391731] Lim AJ Tan E (2024) Social media ills and evolutionary mismatches: a conceptual framework. Evolutionary Psychological Science 10(3): 212–235.

[bibr25-14614448251391731] Lim GJRY Wu SL Yap CC , et al. (2023) Parenting practices and adolescents’ mental health: serial mediation by parental acceptance and adolescents’ grit. Vulnerable Children and Youth Studies. Epub ahead of print 17 November. DOI: 10.1080/17450128.2023.2282573.

[bibr26-14614448251391731] Linzer DA Lewis JB (2011) PoLCA: an R package for polytomous variable latent class analysis. Journal of Statistical Software 42(10): 1–29.

[bibr27-14614448251391731] Livingstone S Ólafsson K Helsper EJ , et al. (2017) Maximizing opportunities and minimizing risks for children online: the role of digital skills in emerging strategies of parental mediation: maximizing opportunities and minimizing risks. Journal of Communication 67(1): 82–105.

[bibr28-14614448251391731] Lou J Wang M Xie X , et al. (2024) The association between family socio-demographic factors, parental mediation and adolescents’ digital literacy: a cross-sectional study. BMC Public Health 24(1): 2932. 10.1186/s12889-024-20284-4 PMC11515667 39438851

[bibr29-14614448251391731] Lukavská K Hrabec O Lukavský J , et al. (2022) The associations of adolescent problematic internet use with parenting: a meta-analysis. Addictive Behaviors 135: 107423. 10.1016/j.addbeh.2022.107423 35933287

[bibr30-14614448251391731] Marciano L Albanese E Viswanath K , et al. (2023) The protective role of social-oriented digital media use in children’s and adolescents’ life satisfaction during the covid-19 pandemic: a latent growth curve model. European Journal of Health Communication 4(1): 1–27.

[bibr31-14614448251391731] Meeus A Beyens I Geusens F , et al. (2018) Managing positive and negative media effects among adolescents: parental mediation matters – But not always. Journal of Family Communication 18(4): 270–285.

[bibr32-14614448251391731] Meyer JP Morin AJS (2016) A person-centered approach to commitment research: theory, research, and methodology. Journal of Organizational Behavior 37(4): 584–612.

[bibr33-14614448251391731] Mori C Temple JR Browne D , et al. (2019) Association of sexting with sexual behaviors and mental health among adolescents: a systematic review and meta-analysis. JAMA Pediatrics 173(8): 770. 10.1001/jamapediatrics.2019.1658 PMC6580450 31206151

[bibr34-14614448251391731] Nagy B Kutrovátz K Király G , et al. (2023) Parental mediation in the age of mobile technology. Children & Society 37(2): 424–451.

[bibr35-14614448251391731] Nielsen P Favez N Liddle H , et al. (2019) Linking parental mediation practices to adolescents’ problematic online screen use: a systematic literature review. Journal of Behavioral Addictions 8(4): 649–663. 10.1556/2006.8.2019.61 PMC7044585 31786936

[bibr36-14614448251391731] Nikken P Jansz J (2014) Developing scales to measure parental mediation of young children’s internet use. Learning, Media and Technology 39(2): 250–266.

[bibr37-14614448251391731] Noom MJ Deković M Meeus WHJ (1999) Autonomy, attachment and psychosocial adjustment during adolescence: a double-edged sword? Journal of Adolescence 22(6): 771–783. 10.1006/jado.1999.0269 10579889

[bibr38-14614448251391731] Nylund-Gibson K Garber AC Carter DB , et al. (2023) Ten frequently asked questions about latent transition analysis. Psychological Methods 28(2): 284–300. 10.1037/met0000486 35834194

[bibr39-14614448251391731] Opgenhaffen M Vandenbosch L Eggermont S , et al. (2012) Parental mediation of television viewing in the context of changing parent-child relationships in Belgium: a latent growth curve analysis in early and middle adolescence. Journal of Children and Media 6(4): 469–484.

[bibr40-14614448251391731] Padilla-Walker LM Stockdale LA McLean RD (2020) Associations between parental media monitoring, media use, and internalizing symptoms during adolescence. Psychology of Popular Media 9(4): 481–492.

[bibr41-14614448251391731] Peng B Hu N Yu H , et al. (2021) Parenting style and adolescent mental health: the chain mediating effects of self-esteem and psychological inflexibility. Frontiers in Psychology 12: 738170. 10.3389/fpsyg.2021.738170 PMC8548717 34721210

[bibr42-14614448251391731] Pinquart M (2016) Associations of parenting styles and dimensions with academic achievement in children and adolescents: a meta-analysis. Educational Psychology Review 28(3): 475–493.

[bibr43-14614448251391731] Przybylski AK Weinstein N (2017) A large-scale test of the Goldilocks hypothesis: quantifying the relations between digital-screen use and the mental well-being of adolescents. Psychological Science 28(2): 204–215. 10.1177/0956797616678438 28085574

[bibr44-14614448251391731] Racine N McArthur BA Cooke JE , et al. (2021) Global prevalence of depressive and anxiety symptoms in children and adolescents during COVID-19: a meta-analysis. JAMA Pediatrics 175(11): 1142–1150. 10.1001/jamapediatrics.2021.2482 PMC8353576 34369987

[bibr45-14614448251391731] Rasmussen EE Rhodes N Ortiz RR , et al. (2016) The relation between norm accessibility, pornography use, and parental mediation among emerging adults. Media Psychology 19(3): 431–454.

[bibr46-14614448251391731] Ren W Zhu X (2022) Parental mediation and adolescents’ internet use: the moderating role of parenting style. Journal of Youth and Adolescence 51(8): 1483–1496. 10.1007/s10964-022-01600-w PMC8978487 35377097

[bibr47-14614448251391731] Ryan RM Deci EL (2000) Self-determination theory and the facilitation of intrinsic motivation, social development, and well-being. American Psychologist 55: 68–78. 10.1037//0003-066x.55.1.68 11392867

[bibr48-14614448251391731] Ryan RM Deci EL (eds) (2017) Self-Determination Theory: Basic Psychological Needs in Motivation, Development, and Wellness. Guilford Press. Available at: https://www.google.com/books/edition/Self_Determination_Theory/th5rDwAAQBAJ?hl=en&gbpv=1&dq=inauthor:%22Richard+M.+Ryan%22 (accessed 14 March 2024).

[bibr49-14614448251391731] Sawyer SM Azzopardi PS Wickremarathne D , et al. (2018) The age of adolescence. The Lancet Child & Adolescent Health 2(3): 223–228. 10.1016/S2352-4642(18)30022-1 30169257

[bibr50-14614448251391731] Scherr S (2022) Social media, self-harm, and suicide. Current Opinion in Psychology 46: 101311. 10.1016/j.copsyc.2022.101311 35339980

[bibr51-14614448251391731] Schroeder RD Mowen TJ (2014) Parenting style transitions and delinquency. Youth & Society 46(2): 228–254.

[bibr52-14614448251391731] Shin W Lwin MO (2022) Parental mediation of children’s digital media use in high digital penetration countries: perspectives from Singapore and Australia. Asian Journal of Communication 32(4): 309–326.

[bibr53-14614448251391731] Soenens B Vansteenkiste M Van Petegem S (2017) Autonomy in Adolescent Development: Towards Conceptual Clarity (ed. Soenens B Vansteenkiste M Van Petegem S ). 1st ed. Abingdon: Routledge. Available at: https://www.taylorfrancis.com/books/9781317265269 (accessed 23 October 2024).

[bibr54-14614448251391731] Tan CY Cheung HS Lee SMS (2025) Parental involvement, parenting styles, and children’s academic outcomes: a second-order, three-level meta-analysis. Review of Educational Research. Epub ahead of print 30 July. DOI: 10.3102/00346543251346792.

[bibr55-14614448251391731] Teuber Z Tang X Sielemann L , et al. (2022) Autonomy-related parenting profiles and their effects on adolescents’ academic and psychological development: a longitudinal person-oriented analysis. Journal of Youth and Adolescence 51(7): 1333–1353. 10.1007/s10964-021-01538-5 PMC9135772 34807340

[bibr56-14614448251391731] Twenge JM Haidt J Joiner TE , et al. (2020) Underestimating digital media harm. Nature Human Behaviour 4(4): 346–348. 10.1038/s41562-020-0839-4 32303719

[bibr57-14614448251391731] Valkenburg PM Piotrowski JT Hermanns J , et al. (2013) Developing and validating the Perceived Parental Media Mediation Scale: a self-determination perspective. Human Communication Research 39(4): 445–469.

[bibr58-14614448251391731] Visser I Speekenbrink M (2010) DepmixS4: an R package for hidden Markov models. Journal of Statistical Software 36(7): 1–21.

[bibr59-14614448251391731] Wang S Chen L Ran H , et al. (2022) Depression and anxiety among children and adolescents pre and post COVID-19: a comparative meta-analysis. Frontiers in Psychiatry 13: 917552. 10.3389/fpsyt.2022.917552 PMC9381924 35990058

[bibr60-14614448251391731] Warren R Aloia L (2019) Parenting style, parental stress, and mediation of children’s media use. Western Journal of Communication 83(4): 483–500.

[bibr61-14614448251391731] West M Rice S Vella-Brodrick D (2023a) Adolescent social media use: cultivating and constraining competence. International Journal of Qualitative Studies on Health and Well-being 18(1): 2277623. 10.1080/17482631.2023.2277623 PMC11000681 37910668

[bibr62-14614448251391731] West M Rice S Vella-Brodrick D (2023b) Mid-adolescents’ social media use: supporting and suppressing autonomy. Journal of Adolescent Research. Epub ahead of print 17 April. DOI: 10.1177/07435584231168402.

[bibr63-14614448251391731] WHO (2022) Mental health. In: Mental Health. Available at: https://www.who.int/news-room/fact-sheets/detail/mental-health-strengthening-our-response (accessed 12 January 2024).

[bibr64-14614448251391731] Wright M (2018) Cyberbullying victimization through social networking sites and adjustment difficulties: the role of parental mediation. Journal of the Association for Information Systems 19: 113–123.

[bibr65-14614448251391731] Wright MF Wachs S (2022) Problematic online gaming, subjective health complaints, and depression among adolescent gamers from the United States: the role of console-gaming aggression. Journal of Children and Media 16(3): 451–460.

