# Supplementary material for: Exploring parental mediation trajectories and their associations with mental health and autonomy in adolescents: A latent transition analysis
Source: New Media Soc. 2025 Nov 20;28(10):5550–70. doi: 10.1177/14614448251391731 (PMC13623226; doi:10.1177/14614448251391731)
Supplement: Supplementary file 1 [file sj-docx-1-nms-10.1177_14614448251391731.docx]

**Appendix A**

*Overview of the parental mediation items entering the latent class analysis at T1*

| **Parental mediation items**  *Original questionnaire items* | *M (SD)* |
| --- | --- |
| **Restrictive mediation**^1^ *During the past year, with respect to the use of computers, tablets, or smartphones, at least one of my parents...* |  |
| *...adjusted the duration of use.* | 1.87 (0.88) |
| *...asked me to put it away during meals.* | 2.32 (0.89) |
| *...asked me to put it away before going to sleep.* | 2.29 (0.90) |
| *...used it as a reward or punishment.* | 1.60 (0.81) |
| **Active mediation**^2^ |  |
| *...talked to me about what I was doing.* | 1.84 (0.82) |
| *...encouraged me to explore and learn things on my own.* | 1.61 (0.78) |
| *...recommended sites to me.* | 1.60 (0.78) |
| *...told me about what happened to me online.* | 1.49 (0.75) |
| *...asked me if I needed any help.* | 1.67 (0.82) |
| *...did activities with me.* | 1.74 (0.82) |
| *...made comments related to activities we did together.* | 1.74 (0.82) |
| **Supervision mediation**^3^ |  |
| *...”glanced over” while I was using devices.* | 1.80 (0.84) |
| *...was present to answer questions if necessary.* | 1.83 (0.87) |
| *...was next door while I was using devices.* | 1.57 (0.78) |
| **Active mediation of Internet safety**^2,4^ |  |
| *...explained to me why some sites/applications are good or harmful.* | 2.02 (0.88) |
| *...suggested how to use the Internet safely.* | 1.99 (0.86) |
| *...helped when I was struggling to do something or look something up on the Internet.* | 1.83 (0.84) |
| *...suggested how to deal with other people online.* | 2.03 (0.90) |
| *...helped when something bothered me on the Internet.* | 1.91 (0.90) |

*Note.* *N* = 717; *M =* mean; *SD =* standard deviation; all items were originally measured using a Likert scale matrix (5-point total) and recoded into low, medium, and high (range 1-3) to improve interpretability and reduce model complexity in subsequent analyses. ^1^Hiniker et al. (2016; ^2^Livingstone et al. (2017); ^3^Nikken & Jansz (2014); ^4^Dürager & Sonck (2014).

**Appendix B**

*Model fit statistics for latent class analysis*

| Time point | Classes | AIC | BIC | aBIC | Entropy |
| --- | --- | --- | --- | --- | --- |
| T1 | 2 | 24281.15 | 24660.88 | 24562.87 | 0.060 |
| **T1** | **3** | **23563.06** | **24148.67** | **23997.52** | **0.114** |
| T1 | 4 | 23092.13 | 23883.62 | 23679.33 | 0.164 |
| T1 | 5 | 22779.28 | 23776.65 | 23519.22 | 0.181 |
| T2 | 2 | 23848.16 | 24227.89 | 24129.88 | 0.061 |
| **T2** | **3** | **22975.47** | **23561.08** | **23409.93** | **0.093** |
| T2 | 4 | 22677.25 | 23468.74 | 23264.46 | 0.149 |
| T2 | 5 | 22431.76 | 23429.13 | 23171.71 | 0.188 |
| T3 | 2 | 21998.68 | 22378.41 | 22280.40 | 0.081 |
| **T3** | **3** | **21494.31** | **22079.92** | **21928.77** | **0.127** |
| T3 | 4 | 21329.94 | 22121.43 | 21917.14 | 0.176 |
| T3 | 5 | 21134.59 | 22131.96 | 21874.53 | 0.209 |

**Appendix C**

*Class share probabilities across time points*

|  | **Probability (*N*)** | | |
| --- | --- | --- | --- |
| *Class* | *T1* | *T2* | *T3* |
| (1) *Enforcing & Engaged Approach* | 0.161 (116) | 0.159 (114) | 0.148 (106) |
| (2) *Hands-off Approach* | 0.443 (317) | 0.375 (269) | 0.458 (328) |
| (3) *Moderate Approach* | 0.396 (284) | 0.466 (334) | 0.395 (283) |

*Note.* Values indicate class proportions, with parentheses showing sample sizes (*N*). *N* = 717.

**Appendix D**

*Class transition probabilities across time points*

|  | **T1**➝**T2** | | | **T2**➝**T3** | | |
| --- | --- | --- | --- | --- | --- | --- |
| *From/to* | *Class 1* | *Class 2* | *Class 3* | *Class 1* | *Class 2* | *Class 3* |
| *Class 1* | 0.555 | 0.294 | 0.151 | 0.501 | 0.354 | 0.145 |
| *Class 2* | 0.304 | 0.395 | 0.300 | 0.092 | 0.620 | 0.288 |
| *Class 3* | 0.189 | 0.473 | 0.338 | 0.116 | 0.687 | 0.198 |

*Note.* Values indicate the probability of transitioning to each class at the next time point, based on prior class membership. Higher values suggest a greater probability of transitioning or remaining in the same class. Class 1 = *Enforcing & Engaged Approach*; Class 2 = *Hands-off Approach*; Class 3 = *Moderate Approach. Grey cells indicate class stability, i.e., the probability of staying in the same class over time.*

**Appendix E**

*Regression results for mental health subdomains (T3)*

| **Mental health subdomain** | **Predictor** | **RC: Enforcing & Engaged Approach** | | | **RC: Moderate Approach** | | | |
| --- | --- | --- | --- | --- | --- | --- | --- | --- |
|  |  | ***B*** | ***SE*** | ***t*** |  | ***B*** | ***SE*** | ***t*** |
| ***Somatic symptoms*** | Intercept | 2.596*** | 0.260 | 9.999 | Intercept | 2.667 ** | 0.213 | 12.511 |
|  | Moderate Approach | 0.071 | 0.159 | 0.444 | Enforcing & Engaged Approach | -0.071 | 0.159 | -0.444 |
|  | Hands-off Approach | 0.008 | 0.157 | 0.051 | Hands-off Approach | -0.063 | 0.080 | -0.781 |
|  | Social desirability | 0.239*** | 0.059 | 4.083 | Social desirability | 0.239*** | 0.059 | 4.083 |
| ***Sleep*** | Intercept | 2.145*** | 0.230 | 9.338 | Intercept | 2.197*** | 0.189 | 11.651 |
|  | Moderate Approach | 0.052 | 0.141 | 0.369 | Enforcing & Engaged Approach | -0.052 | 0.141 | -0.369 |
|  | Hands-off Approach | 0.003 | 0.139 | 0.019 | Hands-off Approach | -0.049 | 0.071 | -0.695 |
|  | Social desirability | 0.385*** | 0.052 | 7.426 | Social desirability | 0.385 ** | 0.052 | 7.426 |
| ***Anxiety*** | Intercept | 1.737*** | 0.286 | 6.067 | Intercept | 1.567*** | 0.235 | 6.668 |
|  | Moderate Approach | -0.170 | 0.175 | -0.968 | Enforcing & Engaged Approach | 0.170 | 0.175 | 0.968 |
|  | Hands-off Approach | -0.152 | 0.173 | -0.879 | Hands-off Approach | 0.018 | 0.088 | 0.202 |
|  | Social desirability | 0.438*** | 0.065 | 6.779 | Social desirability | 0.438*** | 0.065 | 6.779 |
| ***Anger*** | Intercept | 2.265*** | 0.255 | 8.875 | Intercept | 2.227*** | 0.210 | 10.628 |
|  | Moderate Approach | -0.038 | 0.156 | -0.245 | Enforcing & Engaged Approach | 0.038 | 0.156 | 0.245 |
|  | Hands-off Approach | 0.077 | 0.154 | 0.500 | Hands-off Approach | 0.115 | 0.079 | 1.464 |
|  | Social desirability | 0.526*** | 0.058 | 9.121 | Social desirability | 0.526*** | 0.058 | 9.121 |
| ***Inattention*** | Intercept | 2.021*** | 0.215 | 9.413 | Intercept | 1.680*** | 0.176 | 9.535 |
|  | Moderate Approach | **-0.340**** | 0.132 | -2.587 | Enforcing & Engaged Approach | **0.340**** | 0.132 | 2.587 |
|  | Hands-off Approach | **-0.348**** | 0.130 | -2.687 | Hands-off Approach | -0.008 | 0.066 | 0.121 |
|  | Social desirability | 0.511*** | 0.048 | 10.535 | Social desirability | 0.511*** | 0.048 | 10.535 |
| ***OCD*** | Intercept | 2.776*** | 0.305 | 9.112 | Intercept | 2.725*** | 0.250 | 10.895 |
|  | Moderate Approach | -0.051 | 0.187 | -0.273 | Enforcing & Engaged Approach | 0.051 | 0.187 | 0.273 |
|  | Hands-off Approach | 0.068 | 0.184 | 0.369 | Hands-off Approach | 0.119 | 0.094 | 1.262 |
|  | Social desirability | 0.246*** | 0.069 | 3.577 | Social desirability | 0.246*** | 0.069 | 3.577 |
| ***Irritability*** | Intercept | 1.579*** | 0.267 | 5.921 | Intercept | 1.553*** | 0.219 | 7.096 |
|  | Moderate Approach | -0.026 | 0.163 | -0.156 | Enforcing & Engaged Approach | 0.026 | 0.163 | 0.156 |
|  | Hands-off Approach | 0.030 | 0.161 | 0.188 | Hands-off Approach | 0.056 | 0.082 | 0.677 |
|  | Social desirability | 0.574*** | 0.060 | 9.533 | Social desirability | 0.574*** | 0.060 | 9.533 |

*Note. N* = 717; RC = reference class; OCD = obsessive-compulsive disorders; all subdomains were assessed on a 5-point scale: 1 = “never”, 5 = “almost every day”; ***** *p* < .001, ** *p* < .01, ** p* < .05; significant class differences are highlighted in bold

**Appendix C**

*Transition probabilities heatmap*

*Note.* Class 1 *= Enforced & Engaged Approach;* Class 2 *= Hands-off Approach;* Class 3 *= Moderate Approach.*
